# AMR-VMamba: Fine-Grained Image Classification with Adaptive Multi-Level Refinement and Multi-Query Discriminative Pooling

**DOI:** 10.3390/jimaging12100465

**Published:** 2026-09-22

**Authors:** Yiyan Huang, Lei Yang, Zihao Li

**Affiliations:** School of Artificial Intelligence, Tianjin University of Science and Technology, Tianjin 300457, China; 13331303973@163.com (L.Y.); 18257006169@163.com (Z.L.)

**Keywords:** fine-grained image classification, adaptive multi-level refinement, multi-query discriminative pooling, visual state-space model

## Abstract

Fine-grained visual classification (FGVC) requires models to distinguish subtle inter-class differences while remaining robust to substantial intra-class variations in pose and background. Hierarchical visual backbones improve semantic abstraction but progressively compress local textures and part boundaries, while global pooling may further weaken discriminative evidence distributed across multiple regions. To address these issues, we propose AMR-VMamba, a lightweight enhancement framework built on VMamba-Tiny. First, adaptive multi-level refinement (AMR) aligns stage 3 and stage 4 features and selectively injects cross-level differences through position-channel-dependent gating, thereby complementing high-level semantics with mid-level details. Second, multi-query discriminative pooling (MQP) uses a small set of learnable queries to extract multiple local descriptors from the refined 7 × 7 feature grid and combines them with the native pooled representation. Zero-initialized learnable residual coefficients reduce the initial perturbation of the pretrained representation. Across five independent runs on CUB-200-2011, Stanford Dogs, and Oxford Flowers-102, AMR-VMamba achieves Top-1 accuracies of 86.98 ± 0.18%, 88.51 ± 0.26%, and 96.32 ± 0.23%, improving the VMamba-Tiny baseline by 3.05, 0.91, and 1.60 percentage points, respectively, with only 0.5 M additional parameters and 0.02 GMACs.

## 1. Introduction

Fine-grained visual classification (FGVC) aims to distinguish closely related subcategories within the same super-category, such as bird species, dog breeds, and flower categories. Compared with conventional image classification, FGVC is characterized by small inter-class differences and substantial intra-class variations caused by pose, scale, occlusion, and background changes. Discriminative cues are often concentrated in limited regions involving textures, edges, local contours, or part morphology. Consequently, an effective model should capture broad contextual relationships while preserving local details that remain informative throughout hierarchical semantic abstraction.

Existing studies have improved the extraction and organization of local discriminative information through region matching, multi-level feature fusion, and attention modeling. Liu et al. enhanced class separability in ultra-fine-grained recognition through region alignment and proxy learning [1]; Black and Souvenir improved local structural comparison in fine-grained few-shot classification through part matching [2]; and Jia et al. combined self-supervised learning with multi-level feature fusion to jointly exploit shallow details and deep semantics [3]. Guo et al. further integrated spatial- and frequency-domain information [4], whereas Yang et al. used significant feature suppression and cross-feature fusion to reduce over-reliance on a single salient region [5]. These studies indicate that fine-grained recognition depends not only on high-level semantics but also on whether local information can be effectively preserved and aggregated.

State-space models have recently provided a new route for efficient long-range dependency modeling. Mamba introduced a selective state-space mechanism for efficient sequence modeling [6], and VMamba extended this idea to visual grids through two-dimensional selective scanning [7]. Subsequent models, including MambaVision, PVMamba, and DefMamba, improved visual state-space modeling through hybrid architectures, dynamic state aggregation, and deformable scanning, respectively [8,9,10]. Chen et al. also investigated Vision Mamba distillation for low-resolution fine-grained image classification [11]. These developments demonstrate the strong contextual modeling capability of visual state-space models, while fine-grained classification still requires better preservation of hierarchical details and more effective aggregation of discriminative evidence at the classification head.

In this work, VMamba-Tiny is adopted as the baseline. For a 224 × 224 input image, stage 3 retains a 14 × 14 spatial grid and therefore preserves relatively dense local textures, edges, and part structures, whereas stage 4 is further downsampled to 7 × 7 and produces a higher-level semantic representation close to the classification head. The native classification pathway mainly aggregates the highest-stage feature, so discriminative mid-level information from stage 3 is not explicitly incorporated into the final representation. In addition, a single global pooling operation compresses responses from all spatial positions into one descriptor, which may weaken local evidence distributed across multiple regions.

To address these limitations, we propose AMR-VMamba. First, adaptive multi-level refinement (AMR) aligns the stage 3 and stage 4 features in spatial and channel dimensions and applies a position-channel-dependent gate to selectively weight their cross-level difference, allowing mid-level details to complement the high-level semantic representation through residual injection. Second, multi-query discriminative pooling (MQP) employs multiple learnable queries to read several local descriptors from the refined spatial feature grid and fuses the resulting information with the native pooled representation. To reduce the initial disturbance introduced by randomly initialized branches, AMR and MQP are connected through zero-initialized learnable residual coefficients *γ* and *β*, respectively, so that the added pathways contribute progressively during optimization.

The main contributions of this work are summarized as follows:

- An adaptive multi-level refinement module is introduced to selectively fuse the stage 3 and stage 4 features of VMamba-Tiny through position-channel-dependent gating, enabling controlled supplementation of high-level semantics with mid-level local details.
- A multi-query discriminative pooling module is designed to extract multiple spatial descriptors using a small number of learnable queries. Without part-level supervision or full spatial self-attention, MQP supplements the native global representation with distributed local discriminative information.
- AMR and MQP are integrated into VMamba-Tiny through lightweight residual pathways without modifying the native SS2D units or backbone structure, providing stable performance gains with only a small increase in model complexity.

On fine-grained image classification, visual state-space models, and multi-level feature fusion and discriminative aggregation. Section 3 presents the architecture and key components of AMR-VMamba. Section 4 describes the experimental setup and reports the results, Section 5 discusses the findings and limitations, and Section 6 concludes the paper and outlines future work.

## 2. Related Work

### 2.1. Fine-Grained Image Classification

The central challenge of FGVC is to extract stable local differences from visually similar categories. Existing methods commonly strengthen discriminative representations through region localization, local matching, multi-level feature fusion, or attention modeling. Liu et al. improved category separation in ultra-fine-grained novel class discovery through region alignment and proxy learning [1]; Black and Souvenir established local correspondences between support and query samples through part matching [2]; Jia et al. fused features from different Transformer layers to jointly exploit color, texture, contour, and deep semantic information [3]; Guo et al. enhanced fine-grained representations through spatial-frequency feature fusion [4]; and Yang et al. explored complementary discriminative regions using significant feature suppression and cross-feature fusion [5]. More recently, Yuan et al. proposed FGBNet [12], a ConvNeXt-based fine-grained classification network that combines detail-information supplementation, multi-level feature interaction, and Coordinate Attention.

These studies demonstrate that different spatial regions and feature levels provide complementary information for FGVC. Existing approaches commonly emphasize region matching, multi-branch feature interaction, frequency-domain supplementation, or salient-response reweighting. By contrast, AMR treats intermediate-stage information as a controlled correction to the high-level representation. Specifically, after spatial and channel alignment, it explicitly models the cross-level difference, modulates this difference with a position-channel-dependent gate, and injects the selected information through a residual pathway. This design enables AMR to regulate both the content and strength of cross-level compensation, rather than directly concatenating or adding intermediate features.

### 2.2. Visual State-Space Models

Mamba achieves linear-complexity sequence modeling through selective state-space mechanisms [6], and VMamba extends state-space computation to visual grids using two-dimensional selective scanning [7]. Building on this direction, MambaVision combines Mamba and Transformer components [8], PVMamba improves parallel visual Mamba modeling through dynamic state aggregation [9], and DefMamba introduces deformable scanning to enhance spatial adaptability [10]. MambaOut further examines whether Mamba-like structures are always necessary for vision tasks [13], while TinyViM studies lightweight visual Mamba from a frequency-decoupling perspective [14].

For fine-grained recognition, Chen et al. employed a Vision Mamba teacher model for low-resolution fine-grained image classification distillation [11]. In contrast, our method does not modify the SS2D unit, scanning strategy, or backbone organization of VMamba-Tiny. Instead, it performs an incremental enhancement on the hierarchical features already produced by the backbone, focusing on how stage 3 local information can complement stage 4 semantics and how spatial evidence can be aggregated more effectively at the classification head.

### 2.3. Multi-Level Feature Fusion and Discriminative Information Aggregation

Features at different depths of a hierarchical vision network exhibit distinct properties: intermediate representations generally preserve richer textures, edges, and local contours, whereas high-level representations provide stronger semantic abstraction. Jia et al. demonstrated the complementarity of multi-level features [3], and Yang et al. further enhanced interactions across feature stages through cross-feature fusion [5]. Nevertheless, direct concatenation or addition does not guarantee that all intermediate information is useful, indicating that both the content and the strength of cross-level fusion should be controlled.

Global average pooling compresses all spatial positions into a single descriptor. When discriminative evidence is distributed over multiple local regions, such compression can weaken informative responses. Part matching [2] and significant feature suppression [5] both support the value of explicitly modeling multiple local regions. Accordingly, MQP uses a small set of learnable queries to read multiple local descriptors from a 7 × 7 feature grid without constructing full token-to-token self-attention or requiring part-level supervision.

Based on these observations, AMR-VMamba integrates cross-level refinement and spatial aggregation within one framework: AMR selectively compensates stage 4 semantics with information from stage 3, MQP reads multiple spatially discriminative descriptors, and the learnable coefficients *γ* and *β* control the contribution of the newly introduced pathways. The native four-stage VMamba-Tiny backbone and its SS2D units remain unchanged, with structural modifications limited to the cross-level feature interface and the classification aggregation interface.

## 3. Materials and Methods

### 3.1. VMamba-Tiny Baseline and Two-Dimensional Selective State-Space Modeling

AMR-VMamba is built directly on VMamba-Tiny [7]. The original stem, stages 1–4, inter-stage downsampling, SS2D computation, and classifier connection are retained, while the stage 3 feature, stage 4 feature, and native pooled representation are additionally extracted for subsequent refinement and discriminative aggregation. For a 224 × 224 input image, the four stages have approximate spatial resolutions of 56 × 56, 28 × 28, 14 × 14, and 7 × 7. Because channel configurations can vary across VMamba-Tiny implementations, the stage widths are denoted as C1–C4, with the actual tensor dimensions determined by the implementation used in the experiments.

A core component of VMamba is Selective Scan 2D (SS2D). Unlike global self-attention, which explicitly models pairwise relationships among tokens, SS2D rearranges a two-dimensional feature map into one-dimensional sequences along predefined scanning directions, propagates contextual information through selective state-space updates, and then restores and merges the directional outputs in the original spatial layout. For a single scan sequence, *x_t_*, *h_t_*, and *y_t_* denote the input, hidden state, and output at position *t*, respectively; A denotes the state-transition matrix; and *B_t_*, *C_t_*, and the step size Δ*_t_* can be dynamically modulated by the input. The state update and two-dimensional scanning process are expressed as follows [7]:

(1) $$h_{t}={\bar{A}}_{t}h_{t-1}+{\bar{B}}_{t}x_{t},y_{t}=C_{t}h_{t}+Dx_{t}$$

(2) $${\bar{A}}_{t}=exp(\Delta_{t}A),{\bar{B}}_{t}=\left(exp(\Delta_{t}A)-I\right)A^{-1}B_{t}$$

(3) $$F_{\mathrm{ssm}}=\mathrm{Merge}\left(S_{\to }(F),S_{\leftarrow }(F),S_{↓}(F),S_{↑}\left(F\right)\right)$$

Rather than appending new SS2D blocks or modifying the native state-space operator, this study focuses on the hierarchical features already generated by the pretrained backbone and on the aggregation strategy at the classification head. This design retains the contextual modeling capability of VMamba-Tiny while isolating two questions that are particularly relevant to fine-grained recognition: how complementary information across stages can be exploited and how multiple spatially distributed cues can be aggregated at the classification stage.

Specifically, the stage 3 output *F*_3_ and stage 4 output *F*_4_ are selected as the cross-level inputs. *F*_3_ retains a 14 × 14 grid and therefore denser local sampling, whereas *F*_4_ has a 7 × 7 grid after deeper semantic modeling and directly precedes the classification head. Using two adjacent high-level stages enables complementary interaction between mid-level local information and high-level semantics while avoiding the larger computational overhead associated with 28 × 28 or 56 × 56 auxiliary branches. The architecture of the VMamba-Tiny baseline is shown in Figure 1.

### 3.2. Overall Architecture of AMR-VMamba

Given an RGB input image I, VMamba-Tiny first extracts hierarchical visual features along its native forward pathway and retains the stage 3 feature *F*_3_, the stage 4 feature *F*_4_, and the native global pooled representation z_base_. This process can be written as:

(4) $$F_{3},F_{4},z_{\mathrm{base}}=B\left(I\right)$$

where B(*·*) denotes the pretrained VMamba-Tiny backbone. For a 224 × 224 input, the spatial resolutions of *F*_3_ and *F*_4_ are 14 × 14 and 7 × 7, respectively.

AMR-VMamba then enhances the native representation through two consecutive stages. First, AMR performs lightweight downsampling and channel projection on *F*_3_ and semantic projection on *F*_4_ so that both features are mapped to a common 192 × 7 × 7 space. A position-channel-dependent gate is predicted from the aligned features, and the cross-level difference is selectively weighted and injected as a residual to obtain the refined feature:

(5) $$F_{\mathrm{ref}}=\mathrm{AMR}\left(F_{3},F_{4};\gamma \right)$$

where *γ* is a learnable AMR residual coefficient that controls the effective contribution of mid-level features to the high-level semantic representation. The feature alignment, gating, and residual refinement operations are detailed in Section 3.2.1.

Next, MQP flattens *F*_ref_ into *N* = 49 spatial tokens and uses K learnable queries to adaptively read information from different spatial locations. The resulting local descriptors are aggregated to form:

(6) $$z_{\mathrm{mq}}=\mathrm{MQP}\left(F_{\mathrm{ref}};Q\right),Q=\{q_{1},q_{2},\ldots ,q_{K}\}$$

where Q denotes the set of *K* learnable queries. Compared with a single global pooling operation, this procedure extracts multiple local descriptors and therefore supplies additional spatially distributed discriminative information.

To preserve the native classification representation learned by VMamba-Tiny, the MQP output does not replace *z*_base_; instead, it is projected and fused through a residual connection:

(7) $$z=z_{\mathrm{base}}+tanh\left(\beta \right)\;P\left(z_{\mathrm{mq}}\right)$$

where *P*(·) is a linear projection used for dimensional alignment, *β* is a zero-initialized learnable residual coefficient, and *z* is the final enhanced classification representation. The native classifier is then reused to obtain the category prediction:

(8) $$\hat{y}=\mathrm{Classifier}\left(z\right)$$

Figure 2 illustrates the overall architecture of AMR-VMamba. The method leaves the core SS2D units and the native backbone unchanged, refines adjacent high-level features with AMR, and then aggregates multiple spatial descriptors with MQP. AMR and MQP are connected through the learnable coefficients *γ* and *β*, both initialized to zero, so that the added pathways have limited influence at the beginning of training and progressively learn their effective contributions during optimization.

#### 3.2.1. Adaptive Multi-Level Refinement (AMR)

To exploit complementary information across VMamba-Tiny stages, we design the adaptive multi-level refinement (AMR) module. AMR first aligns stage 3 and stage 4 features in spatial and channel dimensions, then adaptively modulates their cross-level difference through a position-channel-dependent gate, and finally injects the selected information into the high-level representation through a residual pathway.

Because the stage 3 feature *F*_3_ and stage 4 feature F_4_ differ in both spatial resolution and channel dimension, AMR first aligns them. On the stage 3 branch, a 3 × 3 depth-wise convolution with stride 2 reduces the spatial resolution from 14 × 14 to 7 × 7, followed by a 1 × 1 point-wise projection that maps the channel dimension to *C* = 192, yielding the detail feature *F_d_*. On the stage 4 branch, a 1 × 1 projection maps *F*_4_ to the same channel dimension, producing the semantic feature *F_s_*. The alignment process is expressed as:

(9) $$F_{d}=P_{d}\left({DWConv}_{3\times 3,s=2}\left(F_{3}\right)\right),F_{s}=P_{s}\left(F_{4}\right)$$

where *P_d_*(·) and *P_s_*(·) denote the channel projections for the stage 3 and stage 4 branches, respectively. After alignment, *F_d_*,*F_s_* ∈ R^(192×7×7)^, enabling position-wise and channel-wise interaction in a common feature space. Compared with direct interpolation or simple addition, the proposed alignment retains local spatial transformation during downsampling while keeping the added computation concentrated in lightweight convolution and projection operations.

The aligned features *F_s_* and *F_d_* are concatenated along the channel dimension and passed through a 1 × 1 convolution followed by a sigmoid function to generate the position-channel-dependent gate *G*:

(10) $$G=\sigma \left({Conv}_{1\times 1}\left(\left[F_{s};F_{d}\right]\right)\right)$$

where [·;·] denotes channel-wise concatenation and *σ*(·) is the sigmoid function, with *G* ∈ [0, 1]^(192×7×7)^. Because *G* preserves both spatial and channel dimensions, the model can adaptively adjust the compensation strength across positions and channels rather than using a single global fusion weight.

The cross-level compensation signal is further obtained from the difference Δ*F* = *F_d_* − *F_s_* and modulated element-wise by *G*. The residual term *R* and the refined feature *F*_ref_ are defined as:

(11) $$R=G⊙\left(F_{d}-F_{s}\right),F_{ref}=F_{s}+tanh\left(\gamma \right)R$$

where ⊙ denotes element-wise multiplication and *γ* is a learnable scalar. Instead of directly combining *F_d_* and *F_s_* without selection, AMR uses the gate to modulate their difference so that mid-level information is introduced as an incremental correction to the high-level semantic representation.

To reduce the disturbance of the added branch at the beginning of fine-tuning, *γ* is initialized to zero. Consequently, tanh(*γ*) is initially close to zero and *F*_ref_ remains close to *F_s_*. During training, *γ* is updated through backpropagation, while tanh(·) bounds the effective residual coefficient to [−1, 1]. AMR therefore provides a controlled cross-level pathway that supplements high-level semantics with stage 3 local details. The structure of the adaptive multi-level refinement module is shown in Figure 3.

#### 3.2.2. Baseline-Preserving Residual Injection

To preserve the classification representation learned by the pretrained VMamba-Tiny, residual connections are used at both the feature and representation levels so that the added modules act as incremental corrections rather than direct replacements of the native pathway. In the AMR-only configuration, the refined feature *F*_ref_ is globally averaged and projected to the same dimensionality as the native pooled representation *z*_base_:

(12) $$z_{base}={Pool}_{VMamba}\left(F_{4}\right),z_{ref}=GAP\left(F_{ref}\right)$$

The resulting representation is then incorporated as an auxiliary residual term:

(13) $$z_{amr}=z_{base}+tanh\left(\beta \right)P\left(z_{ref}\right)$$

where *β* is a zero-initialized learnable scalar that controls the contribution of the newly introduced representation. This design keeps the initial model close to the pretrained VMamba-Tiny representation and allows the AMR branch to contribute progressively during optimization. The same residual fusion mechanism is also applied to the MQP output so that all enhanced configurations follow the same principle of preserving the native main pathway while incrementally refining the classification representation.

The coefficients *γ* and *β* belong to the newly introduced parameter group and are optimized together with the AMR projections, gating network, and MQP queries using the higher learning rate assigned to new modules. The pretrained VMamba-Tiny backbone is fine-tuned with a lower learning rate. The tanh(·) function bounds both residual coefficients within [−1, 1], preventing unbounded growth of the injection amplitude and providing a controlled fusion mechanism.

#### 3.2.3. Multi-Query Discriminative Pooling (MQP)

Although AMR refines the high-level feature using cross-level information, global average pooling still compresses all spatial positions into a single descriptor and may weaken discriminative evidence distributed across multiple local regions. We therefore introduce multi-query discriminative pooling (MQP), which uses multiple learnable queries to read different local descriptors from the refined spatial feature.

Specifically, *F*_ref_ ∈ R^(192×7×7)^ is flattened into *N* = 49 spatial tokens of dimension *C* = 192, denoted by

(14) $$T=\{T_{1},T_{2},\ldots ,T_{N}\},T_{i}\in R^{C}$$

At the same time, *K* learnable queries are introduced:

(15) $$Q=\{q_{1},q_{2},\ldots ,q_{K}\},q_{k}\in R^{C}$$

For the *k*-th query, scaled dot-product similarity is computed against all spatial tokens and normalized with softmax to obtain the corresponding spatial weights:

(16) $$\alpha_{k,i}=\frac{exp\left(q_{k}^{⊤}T_{i}/\sqrt{C}\right)}{\sum_{j=1}^{N}exp\left(q_{k}^{⊤}T_{j}/\sqrt{C}\right)},k=1,\ldots ,K$$

where *α_k,i_* denotes the response weight of the *k*-th query for the *i*-th spatial location. The local descriptor associated with query *k* is then obtained as a weighted sum of all spatial tokens:

(17) $$z_{k}=\sum_{i=1}^{N}\alpha_{k,i}T_{i}$$

The default configuration uses *K* = 4, providing four independent learnable reading slots over the same spatial feature without assigning them to predefined semantic parts. The query-number ablation in Section 4.6 evaluates *K* = 1, 2, 3, 4, and 5 across all three datasets to examine the sensitivity of this empirical default.

The *K* local descriptors are aggregated by mean pooling to obtain the MQP representation *z*_mq_, which is then fused with the native classification representation through the same residual mechanism:

(18) $$z_{\mathrm{mq}}=\frac{1}{K}\sum_{k=1}^{K}z_{k},z=z_{\mathrm{base}}+tanh\left(\beta \right)P\left(z_{\mathrm{mq}}\right)$$

Here, *P*(·) maps the MQP output to the dimensionality of *z*_base_, and *β* is the zero-initialized learnable residual coefficient. MQP therefore supplements the global representation with multiple locally weighted descriptors while retaining the native pooled representation as the main pathway.

Mean aggregation is adopted by default because it keeps the output dimensionality fixed and avoids a parameter increase that grows linearly with *K*. As an ablation, a concat + projection strategy is also evaluated: the *K* descriptors are concatenated into a *KC*-dimensional vector and projected back to *C* dimensions. The effects of aggregation strategy and query number are examined in Section 4.6. The Structure of multi-query discriminative pooling is shown in Figure 4.

### 3.3. Model Information Flow and End-to-End Implementation

Table 1 summarizes the main tensor interfaces of AMR-VMamba for a 224 × 224 input. Because VMamba-Tiny implementations may differ in channel configuration, feature layout, and block details, the four backbone widths are denoted by *C*_1_–*C*_4_, while the newly introduced AMR and MQP branches use a unified feature dimension of *C* = 192. This notation keeps the method description independent of a particular implementation while preserving the same information flow.

During end-to-end inference, the input image first passes through the native VMamba-Tiny backbone to obtain *F*_3_, *F*_4_, and *z*_base_. AMR spatially and channel-wise aligns *F*_3_ and *F*_4_ and selectively modulates their cross-level difference to produce *F*_ref_. MQP then converts *F*_ref_ into spatial tokens, extracts multiple local descriptors using learnable queries, and aggregates them into *z*_mq_. After projection, *z*_mq_ is fused with *z*_base_ through the residual pathway and passed to the native classifier for prediction.

AMR and MQP are integrated as auxiliary branches without changing the SS2D units or the main forward structure of VMamba-Tiny. Consequently, the baseline, AMR-only, MQP-only, and complete AMR-VMamba configurations share the same backbone definition and differ only in the enabled enhancement modules, improving the consistency of ablation comparisons. The overall training and inference procedure is summarized in Table 2.

## 4. Experiments and Results

### 4.1. Experimental Setup and Training Protocol

To ensure fair comparisons among model configurations, all core experiments use the same VMamba-Tiny pretrained initialization, data splits, and image augmentation strategy, and all models are optimized with AdamW. For baseline configuration A, all parameters are optimized as a single group with an initial learning rate of 3 × 10^−4^. For all enhanced configurations, a grouped learning-rate strategy is adopted to reduce disturbance to the pretrained backbone while allowing the newly introduced parameters to learn more rapidly: the VMamba-Tiny backbone uses an initial learning rate of 1 × 10^−4^, whereas the AMR and MQP parameters use 5 × 10^−4^. All configurations use a batch size of 32, 100 training epochs, and a weight decay of 5 × 10^−2^. The software/hardware environment and key hyperparameters are summarized in Table 3 and Table 4.

All experiments were conducted under Windows 11 using PyTorch 1.13 and Python 3.11.5. Model training was performed on an NVIDIA GeForce RTX 4090 GPU (NVIDIA Corporation, Santa Clara, CA, USA) with a 13th Gen Intel Core i9-13900KF CPU (Intel Corporation, Santa Clara, CA, USA), CUDA 12.6, and 24 GB of system memory.

A warm-up + cosine learning-rate schedule is implemented with using LambdaLR in PyTorch 1.13. The first five epochs use linear warm-up, with the learning rate computed as lr × [10^−3^ + (1 − 10^−3^) × (epoch + 1)/5]. Cosine decay is applied from epoch 6 onward, with a minimum learning rate of 1 × 10^−6^.

All three datasets use the same stochastic training-time augmentation strategy: RandomResizedCrop, random horizontal flipping, RandAugment [15], and Random Erasing [16]. These random augmentations are applied only to training samples, whereas validation and test images use deterministic evaluation preprocessing. Oxford Flowers-102 follows its official train/validation/test split, and the official validation set is used for checkpoint selection. Because CUB-200-2011 and Stanford Dogs provide only official training and test partitions, a seed-specific class-stratified validation subset is constructed from each official training set, reserving approximately 5% of the samples in each class and at least one validation sample per class. This yields 394 validation images for CUB-200-2011 and 600 for Stanford Dogs. Within a given random seed, all compared models use exactly the same train/validation split; different seeds may produce slightly different internal splits. In every run, the checkpoint with the highest validation Top-1 accuracy is selected. The official test sets remain fixed and are never used for training, hyperparameter tuning, or checkpoint selection; they are used only for final evaluation of the selected checkpoint in each run.

### 4.2. Datasets

To evaluate AMR-VMamba across different fine-grained recognition scenarios, experiments are conducted on three public benchmarks: CUB-200-2011, Stanford Dogs, and Oxford Flowers-102. The datasets cover bird species, dog breeds, and flower categories and differ in the number of classes, dataset size, and degree of intra-class variation, providing complementary conditions for assessing local discriminative feature modeling.

CUB-200-2011 contains 200 bird species and 11,788 images, including 5994 training images and 5794 test images in the official split [17]. The dataset exhibits substantial variation in pose, background, and imaging conditions, while category differences are often concentrated in local structures such as the beak, head, wings, and tail. It is therefore widely used to evaluate fine-grained modeling of subtle local cues.

Stanford Dogs contains 120 dog breeds and 20,580 images, with 12,000 images used for training and 8580 for testing [18]. Breed-level differences may involve coat color, ear shape, head contour, and fur texture, while viewpoint, action, and background changes introduce substantial intra-class variation. The dataset therefore provides a challenging setting for evaluating stable discriminative representations under complex appearance changes.

Oxford Flowers-102 contains 102 flower categories and 8189 images [19]. The official split consists of 1020 training images, 1020 validation images, and 6149 test images. Many classes have similar color, petal morphology, texture, and global structure, placing strong demands on fine-grained feature extraction. Unlike CUB-200-2011 and Stanford Dogs, Oxford Flowers-102 provides an independent official validation set, which is used directly for model selection. Dataset statistics are summarized in Table 5.

### 4.3. Evaluation Metrics and Statistical Reporting

Top-1 accuracy is used as the primary evaluation metric and measures the proportion of samples for which the highest-probability prediction matches the ground-truth class. Because all three benchmarks are single-label multi-class classification tasks, Top-1 accuracy directly reflects overall classification performance and is computed as:

(19) $${Acc}_{\mathrm{Top}-1}=\frac{1}{N}\sum_{i=1}^{N}I\left({\hat{y}}_{i}=y_{i}\right)\times 100\%$$

where *N* is the number of test samples, *y_i_* and *ŷ_i_* denote the ground-truth and predicted labels of sample *i*, respectively, and I(·) is the indicator function.

To reduce the influence of a single random training run, each core configuration is repeated with *S* = 5 independent random seeds, and the mean Top-1 accuracy, sample standard deviation, and 95% confidence interval (95% CI) are reported. For CUB-200-2011 and Stanford Dogs, the seed also determines the internal stratified train/validation split, while all models compared under the same seed share the identical split. For results *a*_1_, *a*_2_, …, *a_S_*, the mean and sample standard deviation are computed as:

(20) $$\bar{a}=\frac{1}{S}\sum_{s=1}^{S}a_{s},\sigma =\sqrt{\frac{1}{S-1}\sum_{s=1}^{S}{\left(a_{s}-\bar{a}\right)}^{2}},\;S=5$$

All internally evaluated experimental results are reported as mean ± standard deviation over five independent runs. The 95% confidence interval of the mean is computed using Student’s t distribution with four degrees of freedom. To directly assess the difference between VMamba-Tiny and AMR-VMamba under matched randomness, we additionally conduct a two-sided paired *t*-test using results from the same five seeds and report the 95% confidence interval of the paired difference. Statistical significance is assessed at the 0.05 level. These statistics complement, rather than replace, the absolute Top-1 accuracy comparison.

### 4.4. Ablation Configurations and Comparison Protocol

To isolate the individual and combined contributions of AMR and MQP, four core configurations, A–D, are evaluated on CUB-200-2011, Stanford Dogs, and Oxford Flowers-102. Configuration A is the native VMamba-Tiny baseline. Configuration B adds only AMR and uses global average pooling on *F*_ref_ to construct an auxiliary residual representation. Configuration C retains the native backbone and introduces only MQP with *K* = 4 learnable queries over the projected high-level feature. Configuration D enables both AMR and MQP and constitutes the complete AMR-VMamba. Comparing A–D separately evaluates cross-level refinement, multi-query aggregation, and their joint effect.

To ensure comparable ablation results, A–D use the same augmentation strategy, training budget, model-selection rule, and five-run protocol. Within each random seed and dataset, all configurations use the same data split; only the enabled enhancement modules differ. This design reduces non-structural confounding factors so that performance changes primarily reflect the corresponding module configuration.

Starting from the complete configuration D, two additional factors are examined on all three datasets. Configuration E replaces the default mean aggregation with concat + projection, where *K* local descriptors are concatenated and projected back to a fixed dimension. Configurations *F*_1_–*F*_4_ set *K* = 1, 2, 3, and 5, respectively, and are compared with the default *K* = 4 setting in D to assess sensitivity to query capacity over the tested range of *K* = 1–5.

### 4.5. Overall Comparison on Fine-Grained Benchmarks

Table 6 summarizes the performance of ResNet-50 [20], EfficientNet-B0 [21], ConvNeXt-Tiny [22], Swin-Tiny [23], VMamba-Tiny [7], FGBNet [12], and the proposed AMR-VMamba on CUB-200-2011, Stanford Dogs, and Oxford Flowers-102. AMR-VMamba achieves Top-1 accuracies of 86.98 ± 0.18%, 88.51 ± 0.26%, and 96.32 ± 0.23% on the three datasets, respectively, demonstrating consistently strong performance across diverse fine-grained visual recognition tasks.

On CUB-200-2011, AMR-VMamba achieves 86.98 ± 0.18% Top-1 accuracy, improving VMamba-Tiny from 83.93 ± 0.43% by 3.05 percentage points. The relatively large gain is consistent with the strong dependence of bird-species recognition on localized structures such as the head, beak, wings, and feather texture under substantial pose and background variation. AMR supplements high-level semantics with mid-level details, while MQP aggregates discriminative cues distributed across multiple local regions.

On Stanford Dogs, AMR-VMamba reaches 88.51 ± 0.26%, compared with 87.60 ± 0.32% for VMamba-Tiny, corresponding to an improvement of 0.91 percentage points. Dog-breed recognition is particularly challenging because of high inter-class morphological similarity together with substantial intra-class variation in pose, coat appearance, viewpoint, and individual characteristics. The paired statistical analysis further confirms that the observed improvement is significant at the 0.05 level.

On Oxford Flowers-102, AMR-VMamba achieves 96.32 ± 0.23%, improving the VMamba-Tiny baseline of 94.72 ± 0.78% by 1.60 percentage points. Flower recognition also relies strongly on local petal morphology, texture, color distribution, and central structures, which are well aligned with the proposed cross-level refinement and multi-region aggregation mechanisms. AMR-VMamba also achieves the highest accuracy among the compared methods on this dataset.

Across the five paired runs, the 95% confidence intervals of VMamba-Tiny and AMR-VMamba are [83.40, 84.46]% and [86.76, 87.20]% on CUB-200-2011, [87.20, 88.00]% and [88.19, 88.83]% on Stanford Dogs, and [93.75, 95.69]% and [96.03, 96.61]% on Oxford Flowers-102, respectively. Two-sided paired *t*-tests yield mean improvements of 3.05, 0.91, and 1.60 percentage points, with paired-difference 95% confidence intervals of [2.35, 3.75], [0.23, 1.59], and [0.80, 2.40], and *p*-values of 0.0003, 0.021, and 0.005, respectively. These results confirm statistically significant improvements over VMamba-Tiny across all three datasets under the five-seed protocol.

Overall, AMR-VMamba consistently improves the VMamba-Tiny baseline and remains competitive with the other evaluated architectures, including the recent fine-grained classification method FGBNet [12]. The results also reveal dataset-dependent strengths across different architectures, with AMR-VMamba showing particularly strong performance on Oxford Flowers-102.

The performance gains are achieved with only a small increase in theoretical model complexity: the parameter count increases from 29.6 M to 30.1 M and MACs from 4.81 G to 4.83 G. As shown in Figure 5, AMR-VMamba improves CUB-200-2011 accuracy by 3.05 percentage points with only 0.02 additional GMACs. Practical runtime performance is further analyzed in Section 4.8 to complement the theoretical complexity evaluation.

### 4.6. Ablation Study

To analyze the contribution of each component, configurations A–D first isolate AMR and MQP, after which the aggregation strategy and query number are evaluated. All ablation configurations are evaluated on CUB-200-2011, Stanford Dogs, and Oxford Flowers-102 using the same training protocol and five independent random seeds. The results are summarized in Table 7.

The baseline configuration A achieves 83.93 ± 0.43%, 87.60 ± 0.32%, and 94.72 ± 0.78% on CUB-200-2011, Stanford Dogs, and Oxford Flowers-102, respectively. AMR-only (B) improves the results to 85.46 ± 0.51%, 88.06 ± 0.34%, and 95.50 ± 0.52%, while MQP-only (C) reaches 85.88 ± 0.32%, 88.18 ± 0.29%, and 95.72 ± 0.44%. These independent improvements support the distinct contributions of cross-level refinement and multi-region discriminative aggregation.

Combining both modules (D) further improves performance to 86.98 ± 0.18%, 88.51 ± 0.26%, and 96.32 ± 0.23%, corresponding to gains of 3.05, 0.91, and 1.60 percentage points over the baseline. Configuration D also outperforms AMR-only by 1.52, 0.45, and 0.82 points and MQP-only by 1.10, 0.33, and 0.60 points across the three datasets. These additional gains indicate that AMR and MQP are functionally complementary: AMR refines the high-level feature with selected cross-level detail, while MQP subsequently aggregates multiple discriminative descriptors from the refined representation.

For descriptor aggregation, mean pooling (D) achieves 86.98%, 88.51%, and 96.32%, compared with 85.30%, 88.00%, and 95.41% for concat + projection (E). Mean aggregation therefore provides consistently better performance across the three datasets while retaining a simpler aggregation structure, and is adopted as the default setting.

The query-number analysis shows a consistent trend across all three datasets. As *K* increases from 1 to 4, Top-1 accuracy improves from 85.65% to 86.98% on CUB-200-2011, from 88.10% to 88.51% on Stanford Dogs, and from 95.61% to 96.32% on Oxford Flowers-102. Increasing *K* to 5 results in 86.51%, 88.39%, and 96.08%, respectively, indicating that the benefit begins to saturate beyond *K* = 4. Since all queries operate on the same 49 spatial tokens, additional queries may introduce greater response redundancy, while query-token computation also increases approximately linearly with *K*. Considering both recognition performance and computational cost, *K* = 4 is therefore selected as the default configuration.

Overall, the ablation results consistently demonstrate the independent and complementary contributions of AMR and MQP. They further show that mean aggregation is more effective than the tested concat + projection alternative and that a moderate query capacity provides the best performance within the evaluated range. Figure 6 visualizes the component and query-number ablations on CUB-200-2011, while Table 7 reports the corresponding results across all three datasets.

### 4.7. Validation-Loss Convergence Analysis

To examine optimization behavior, Figure 7 compares the validation-loss curves of the VMamba-Tiny baseline and AMR-VMamba on CUB-200-2011. Both models use the same data split, number of training epochs, and augmentation strategy to ensure comparable convergence trends.

For both VMamba-Tiny and AMR-VMamba, validation loss decreases rapidly during the early training stage, after which the fluctuations become smaller and the curves gradually stabilize. Neither model exhibits obvious loss divergence under the adopted optimization protocol.

Although AMR-VMamba introduces both cross-level refinement and multi-query discriminative aggregation on top of pretrained VMamba-Tiny, its validation loss still shows a stable decreasing and convergent trend. This indicates that the added enhancement branches do not adversely disrupt the optimization of the baseline. Together with the zero-initialized residual pathways, the new modules can progressively participate in representation learning during training.

Combined with the classification results, AMR-VMamba improves CUB-200-2011 Top-1 accuracy from 83.93 ± 0.43% to 86.98 ± 0.18% while maintaining stable validation-loss convergence.

### 4.8. Complexity and Efficiency

In addition to classification performance, theoretical complexity and measured hardware behavior are both important for characterizing practical efficiency. Table 8 reports parameter counts and MACs for the ablation configurations and additionally reports single-image inference latency, throughput, and peak GPU memory for VMamba-Tiny and the complete AMR-VMamba under the same RTX 4090 environment and 224 × 224 input resolution.

VMamba-Tiny contains 29.6 M parameters and requires 4.81 GMACs, while the complete AMR-VMamba uses 30.1 M parameters and 4.83 GMACs. The AMR-only and MQP-only variants require 29.7 M/4.83 GMACs and 29.9 M/4.82 GMACs, respectively. Thus, the complete model introduces only 0.5 M additional parameters (+1.69%) and 0.02 GMACs (+0.42%) over VMamba-Tiny.

In practical inference, latency increases from 6.35 to 6.82 ms (+7.4%), throughput changes from 157.5 to 146.6 FPS (−6.9%), and peak GPU memory increases from 1.72 to 1.81 GB (+5.2%). The difference between theoretical and measured overhead reflects the influence of additional projection, gating, memory-access, and GPU execution operations that are not fully captured by MACs. Nevertheless, AMR-VMamba maintains high-throughput inference with only limited additional resource consumption.

Average training time per epoch increases from 20.3 to 22.6 s on CUB-200-2011, from 60.9 to 68.6 s on Stanford Dogs, and from 10.2 to 11.1 s on Oxford Flowers-102. Together with the classification results in Table 6, these measurements indicate that the proposed modules achieve improved fine-grained recognition performance with a relatively small increase in computational and runtime costs.

### 4.9. Robustness to Backbone Capacity

To examine whether the observed benefit extends beyond VMamba-Tiny, we further evaluate the same AMR/MQP design on the higher-capacity VMamba-Small backbone using CUB-200-2011. The stage 3/stage 4 refinement strategy is retained, with both stages projected into a unified 192-dimensional space before AMR and MQP processing. As shown in Table 9, we compare the baseline and AMR/MQP-enhanced configurations under both VMamba-Tiny and VMamba-Small backbones to assess whether the pro-posed design remains effective across different backbone capacities.

AMR/MQP improves VMamba-Tiny from 83.93% to 86.98% (+3.05 points) and VMamba-Small from 85.74% to 87.62% (+1.88 points). The consistent improvements across both backbone scales indicate that the proposed design is not tied to the specific capacity or native channel configuration of VMamba-Tiny, as the unified projection space can accommodate different stage widths. The smaller gain with VMamba-Small may reflect the stronger baseline representation, which already captures a larger portion of the semantic and local discriminative information targeted by AMR and MQP.

## 5. Discussion

AMR-VMamba enhances VMamba through two complementary mechanisms rather than through two redundant attention blocks. AMR differs from conventional direct multi-level fusion because it does not simply concatenate or add intermediate and high-level features. Instead, it aligns stage 3 and stage 4 features, explicitly models their cross-level difference, and selectively injects that difference through a position- and channel-dependent gate, so stage 3 information acts as a controlled correction to stage 4 semantics. The AMR-only configuration improves the baseline on all three datasets, supporting the practical value of this controlled cross-level refinement.

MQP operates at a different stage of the information flow. Rather than replacing global pooling with full self-attention, it uses a small number of learnable queries to read multiple descriptors from the refined 7 × 7 feature map and then supplements the native pooled representation through residual fusion. MQP-only independently improves the baseline, and the complete model provides a further gain over both single-module variants. The expanded *K* = 1–5 ablation shows that performance rises up to *K* = 4 and then decreases slightly at *K* = 5 on all three datasets, indicating that more queries are not unconditionally better.

The magnitude of the improvement varies across datasets. AMR-VMamba achieves the largest gain on CUB-200-2011 (+3.05 points), followed by Oxford Flowers-102 (+1.60 points) and Stanford Dogs (+0.91 points). These differences may be related to dataset-specific fine-grained characteristics and baseline performance. Notably, the improvement on Stanford Dogs remains statistically significant (*p* = 0.021), indicating a consistent gain across the evaluated runs.

The VMamba-Small experiment further shows that the proposed design remains effective with a higher-capacity backbone, improving CUB-200-2011 accuracy from 85.74 ± 0.36% to 87.62 ± 0.24%. The consistent improvements with both VMamba-Tiny and VMamba-Small suggest that AMR and MQP are applicable across different VMamba model capacities.

The comparison with FGBNet further highlights the complementary strengths of AMR-VMamba. FGBNet performs better on CUB-200-2011 and Stanford Dogs, whereas AMR-VMamba achieves higher accuracy on Oxford Flowers-102. Unlike FGBNet, AMR-VMamba preserves the native VMamba backbone and introduces lightweight cross-level refinement and multi-query aggregation without modifying the underlying SS2D units. Future work will explore more flexible stage selection, query configurations, and additional visual state-space backbones.

## 6. Conclusions

This paper proposes AMR-VMamba to address two limitations of VMamba-Tiny for fine-grained image classification: insufficient explicit use of mid-level local details and excessive compression of spatially distributed discriminative evidence by a single global pooling operation. AMR performs controlled cross-level interaction between stage 3 and stage 4 using position-channel-dependent gating, while MQP extracts multiple local descriptors from the refined feature using learnable queries. Zero-initialized residual coefficients *γ* and *β* allow the added pathways to participate progressively in optimization while preserving the native representation as the main pathway.

AMR-VMamba achieves Top-1 accuracies of 86.98 ± 0.18%, 88.51 ± 0.26%, and 96.32 ± 0.23% on CUB-200-2011, Stanford Dogs, and Oxford Flowers-102, respectively, corresponding to improvements of 3.05, 0.91, and 1.60 percentage points over VMamba-Tiny across five independent runs. Paired statistical tests further confirm that these improvements are significant under the adopted experimental protocol (*p* = 0.0003, 0.021, and 0.005, respectively). Expanded ablation studies across all three datasets consistently verify the individual effectiveness and complementary contributions of AMR and MQP. The query-number analysis further shows that *K* = 4 with mean aggregation achieves the best overall performance among the evaluated *K* = 1−5 configurations. The complete model contains 30.1 M parameters and requires 4.83 GMACs, while practical inference measurements indicate only limited additional runtime and memory costs. Taken together, these results demonstrate that AMR-VMamba provides a favorable balance between recognition accuracy and computational efficiency.

Experiments with the higher-capacity VMamba-Small backbone further demonstrate the applicability of the proposed design across different model scales. Using the same AMR and MQP configuration improves CUB-200-2011 accuracy from 85.74 ± 0.36% to 87.62 ± 0.24%, yielding an additional gain of 1.88 percentage points over the stronger baseline. The consistent improvements observed with both VMamba-Tiny and VMamba-Small suggest that the proposed cross-level refinement and multi-query aggregation mechanisms can effectively complement VMamba representations across different backbone capacities. Future work will further explore adaptive cross-level feature selection, alternative projection dimensions and query configurations, and visualization of gate and query responses to better characterize the learned spatial behavior. Extending the framework to additional fine-grained benchmarks and other visual state-space architectures will also provide a broader evaluation of its applicability and scalability.

## Figures and Tables

**Figure 1:**
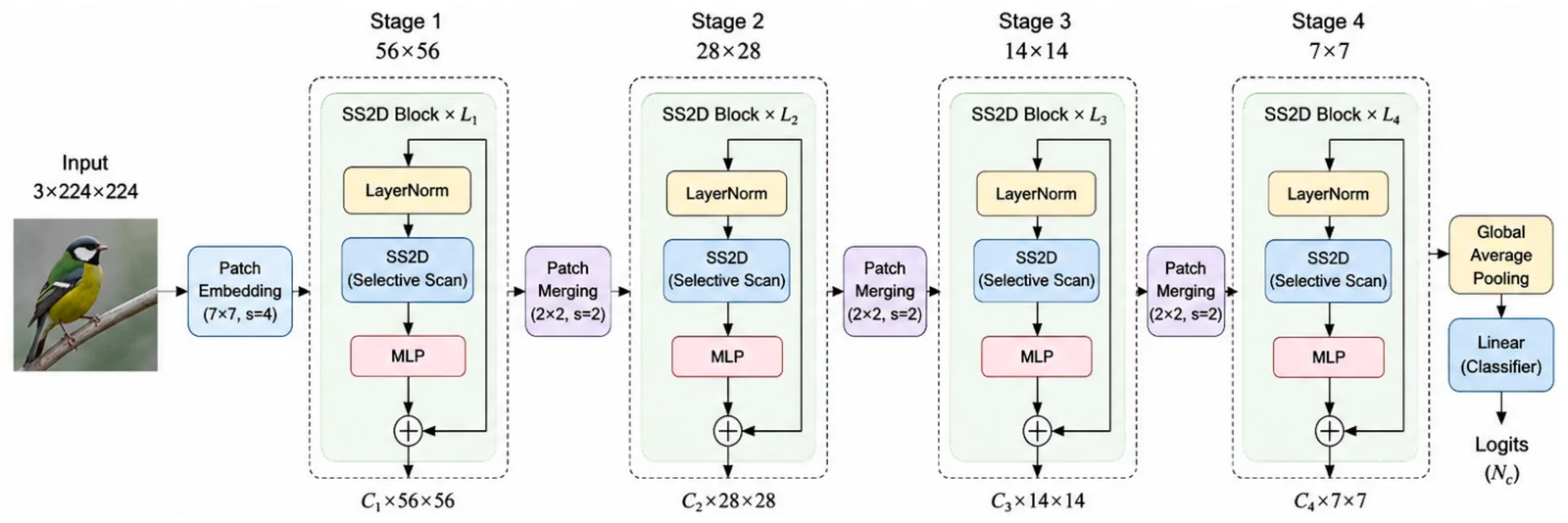
Architecture of the VMamba-Tiny baseline. SS2D denotes Selective Scan 2D, the native state-space operator used within the VMamba stages.

**Figure 2:**
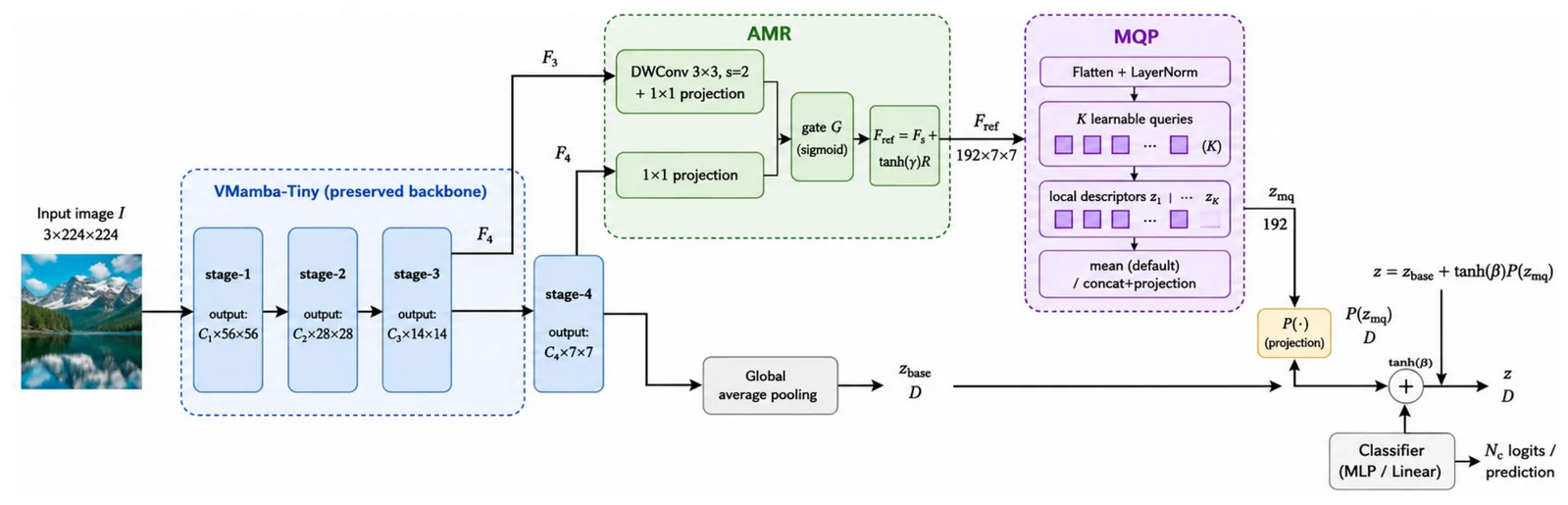
Overall architecture of AMR-VMamba. *F*_3_ and *F*_4_ denote the stage 3 and stage 4 features of VMamba, respectively; *z*_base_ is the native globally pooled representation. Adaptive multi-level refinement (AMR) produces the refined feature *F*_ref_ through gated cross-level correction, and multi-query discriminative pooling (MQP) uses *K* learnable queries to obtain multiple local descriptors. The zero-initialized residual coefficients *γ* and *β* control feature-level and representation-level injection, respectively.

**Figure 3:**
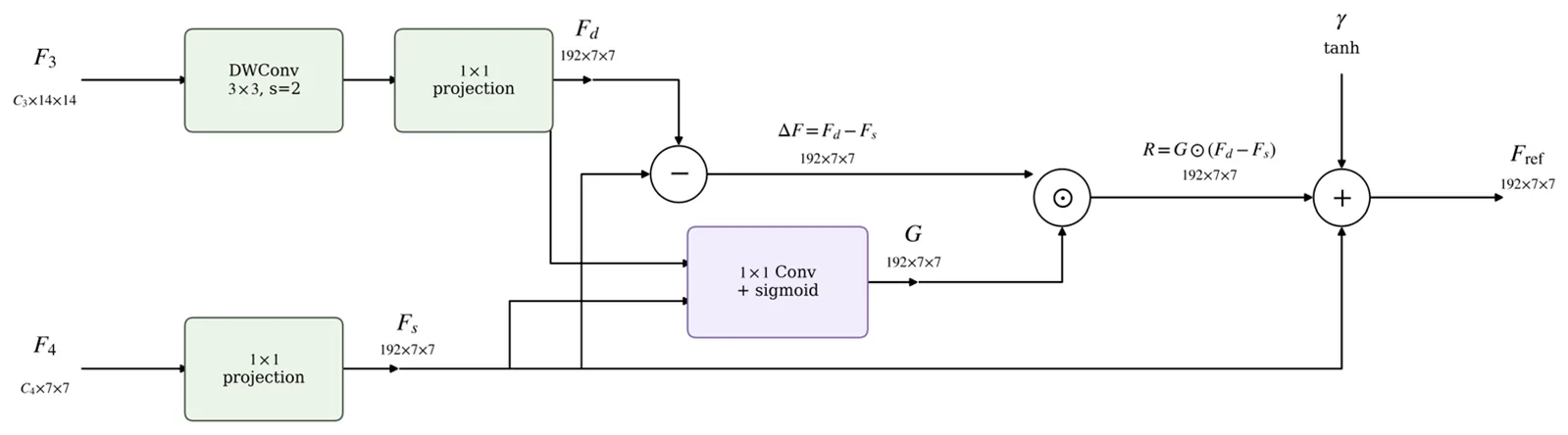
Structure of the adaptive multi-level refinement (AMR) module. *F*_3_ and *F*_4_ denote the stage 3 and stage 4 features, respectively. After spatial and channel alignment, a position-channel-dependent gate adaptively weights their cross-level difference, while the learnable coefficient *γ* controls residual injection into the refined feature *F*_ref_.

**Figure 4:**
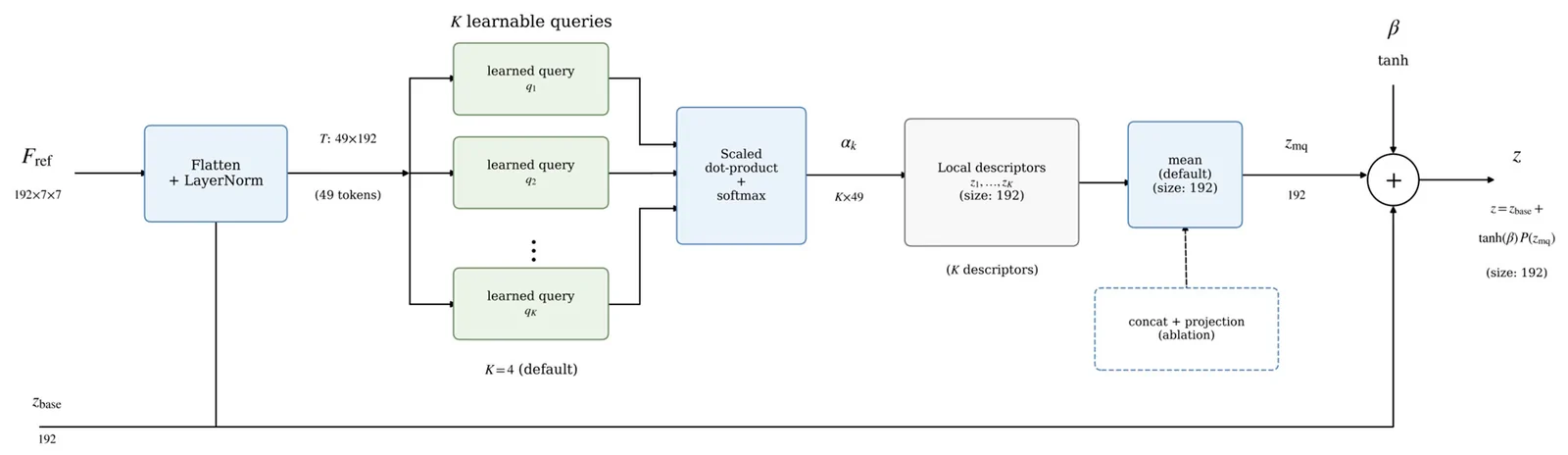
Structure of multi-query discriminative pooling (MQP). The refined feature *F*_ref_ is flattened into *N* = 49 spatial tokens, and *K* learnable queries read the tokens in parallel to form multiple local descriptors. Mean pooling is used as the default aggregation strategy, and the zero-initialized coefficient *β* controls residual fusion with the native pooled representation *z*_base_.

**Figure 5:**
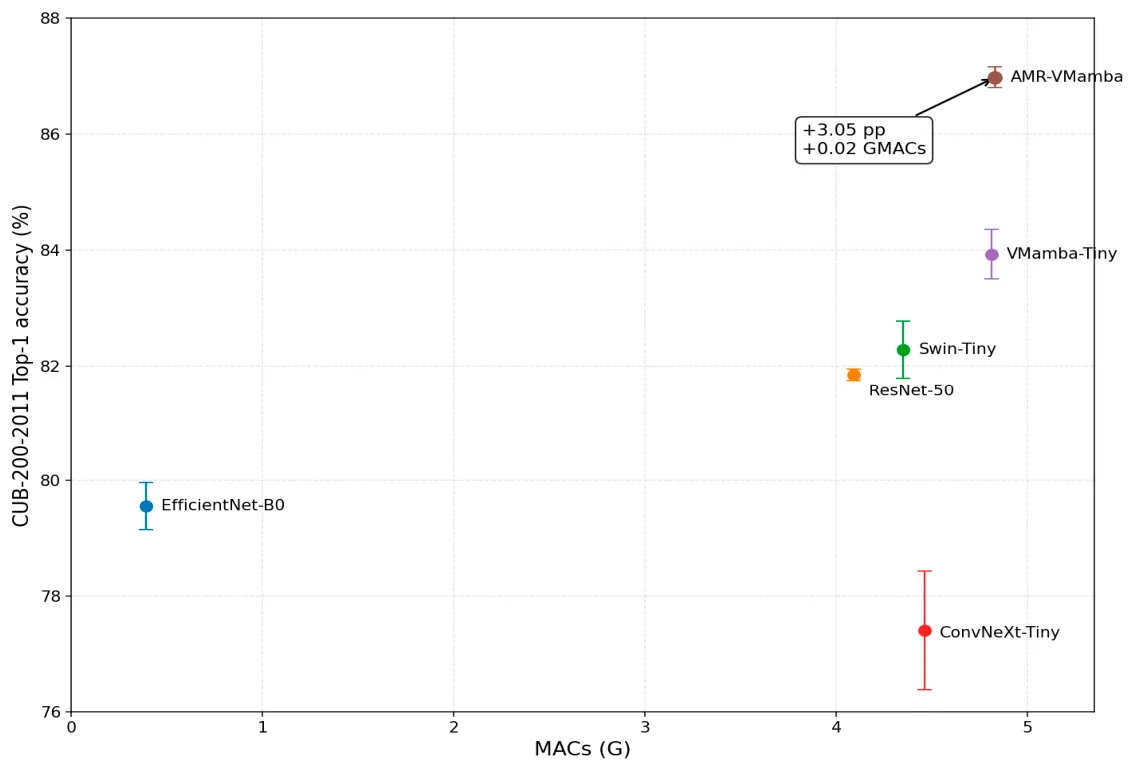
Relationship between Top-1 accuracy and theoretical computational complexity on CUB-200-2011. AMR-VMamba achieves 86.98 ± 0.18% at 4.83 GMACs, improving VMamba-Tiny by 3.05 percentage points with 0.02 additional GMACs. MACs denote multiply-accumulate operations.

**Figure 6:**
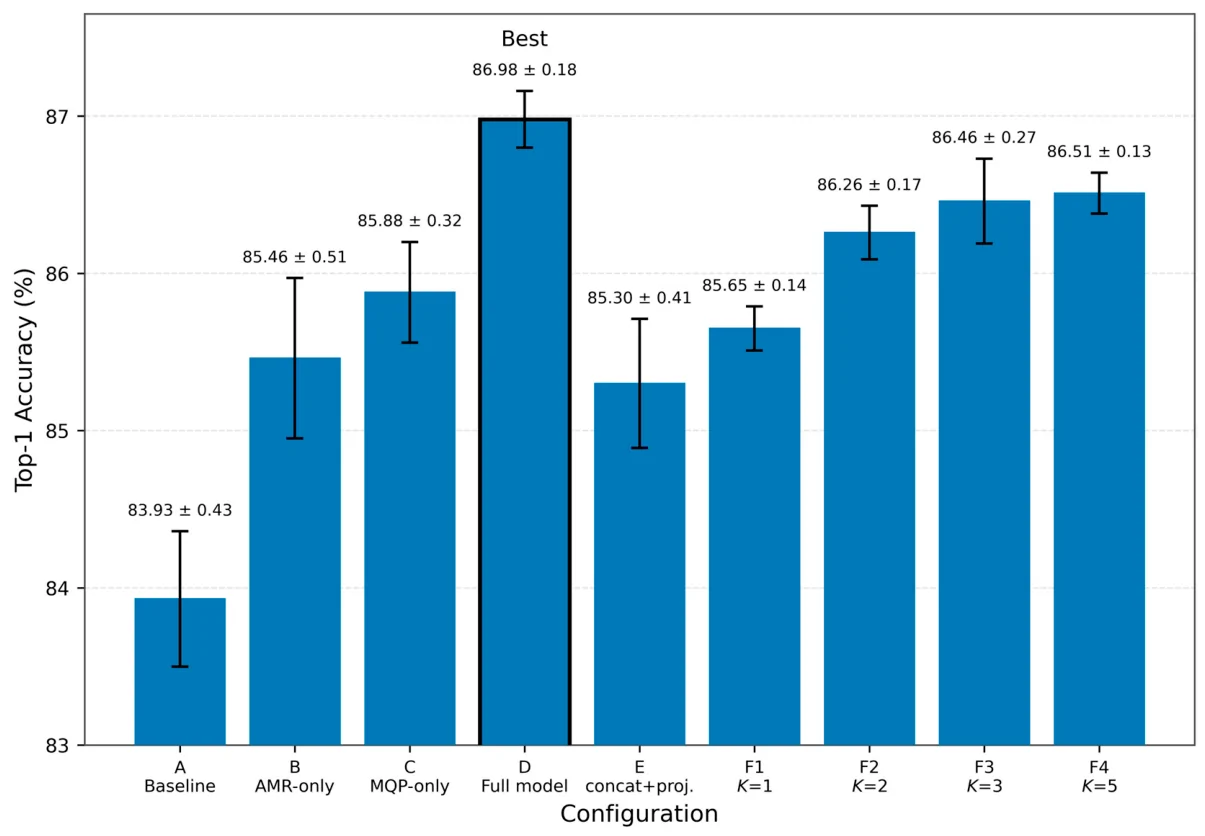
AMR/MQP component and query-number ablation on CUB-200-2011. AMR and MQP denote adaptive multi-level refinement and multi-query discriminative pooling, respectively. D is the complete model with *K* = 4 and mean aggregation; E uses concat + projection; F1–F4 use *K* = 1, 2, 3, and 5. The complete configuration D achieves the best CUB-200-2011 Top-1 accuracy among the tested settings.

**Figure 7:**
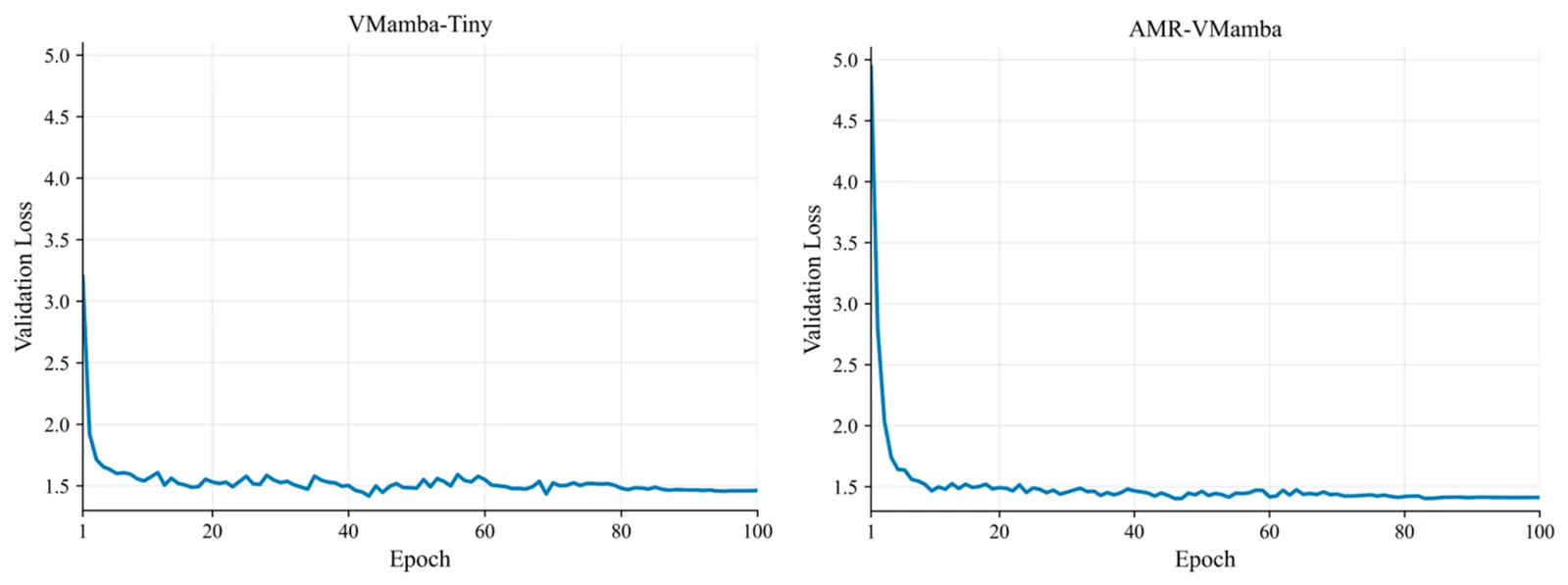
Validation-loss convergence on CUB-200-2011: VMamba-Tiny and AMR-VMamba. The validation loss of both models decreases during training and approaches a stable level in the later epochs.

**Table 1:** Main modules, operations, and tensor dimensions of AMR-VMamba for a 224 × 224 input.

Module/Stage	Main Input	Core Operation	Output Size
Input image	RGB image	Normalization/data augmentation	3 × 224 × 224
VMamba stage 1	3 × 224 × 224	Stem + SS2D blocks	*C*_1_ × 56 × 56
VMamba stage 2	*C*_1_ × 56 × 56	Downsampling + SS2D blocks	*C*_2_ × 28 × 28
VMamba stage 3	*C*_2_ × 28 × 28	Downsampling + SS2D blocks	*C*_3_ × 14 × 14
VMamba stage 4	*C*_3_ × 14 × 14	Downsampling + SS2D blocks	*C*_4_ × 7 × 7
Detail downsampling	*F*_3_	DWConv 3 × 3, *s* = 2	*C*_3_ × 7 × 7
Detail projection	*C*_3_ × 7 × 7	1 × 1 projection	192 × 7 × 7
Semantic projection	*F*_4_	1 × 1 projection	192 × 7 × 7
AMR gate	[*F_s_*; *F_d_*]	1 × 1 Conv + sigmoid	192 × 7 × 7
Difference residual	*F_s_*, *F_d_*, *G*	*G* ⊙ (*F_d_* − *F_s_*)	192 × 7 × 7
Refined feature	*F_s_*, *R*	*F_s_* + tanh(*γ*)R	192 × 7 × 7
MQP tokens	*F*_ref_	Flatten + LayerNorm	49 × 192
Multi-query reading	49 × 192	*K* = 4 learnable queries + scaled dot-product	4 × 192
MQP aggregation	4 × 192	Mean/concat + projection	192
Representation fusion	*z*_base_, *z*_mq_	*z*_base_ + tanh(*β*)*P*(*z*_mq_)	*D*
Classifier	*z*	Linear classifier	*N_c_* logits

where *C*_1_–*C*_4_ denote the channel dimensions of the four VMamba-Tiny stages, *D* denotes the dimensionality of the native classification representation, and *N_c_* is the number of classes.

**Table 2:** Training and inference procedure of AMR-VMamba.

Step	Operation
1	Input an RGB image I and label *y*. During training, use the same RandomResizedCrop, random horizontal flipping, RandAugment [15], and Random Erasing [16] as in the baseline.
2	Forward the image through pretrained VMamba-Tiny and retain the stage 3 feature *F*_3_, stage 4 feature *F*_4_, and native pooled feature *z*_base_.
3	Apply a 3 × 3 DWConv with stride 2 and a 1 × 1 projection to *F*_3_ to obtain *F_d_*; apply a 1 × 1 projection to *F*_4_ to obtain *F_s_*.
4	Predict gate *G* from [*F_s_*; *F_d_*], compute *R* = *G* ⊙ (*F_d_* − *F_s_*), and *F*_ref_ = *F*_s_ + tanh(*γ*)R, where *γ* is initialized to 0.
5	For AMR-only, globally average *F*_ref_ and project it to *z*_ref_; construct *z* = *z*_base_ + tanh(*β*)*P*(*z*_ref_).
6	For MQP, flatten *F*_ref_ and apply LayerNorm to obtain 49 spatial tokens; use *K* learnable queries with scaled dot-product and softmax to compute spatial weights and descriptors *z*_1_,…,*z_K_*.
7	Aggregate the descriptors by mean pooling to obtain *z*_mq_; use concat + projection in the ablation setting. Fuse with the native representation through *z* = *z*_base_ + tanh(*β*)*P*(*z*_mq_), where *β* is initialized to 0.
8	Feed *z* to the classifier to obtain *N*_c_ logits and optimize end-to-end with cross-entropy loss. Use separate learning rates for the pretrained backbone and new modules.
9	Record training/validation loss, Top-1 accuracy, learning rate, *γ*, and *β* during training, and select the final checkpoint using validation performance.
10	During inference, disable stochastic training augmentation and gradient computation and output class probabilities and the final prediction.

**Table 3:** Software and hardware environment used for model training.

Item	Configuration
Operating system	Windows 11
Deep learning framework	PyTorch 1.13
CUDA	12.6
Programming language	Python 3.11.5
CPU	13th Gen Intel Core i9-13900KF
GPU	NVIDIA GeForce RTX 4090
RAM	24 GB

**Table 4:** Main training hyperparameters of AMR-VMamba.

Hyperparameter	Configuration	Scope/Description
Optimizer	AdamW	All configurations
Batch size	32	All configurations
Epochs	100	All configurations
Weight decay	5 × 10^−2^	All parameter groups

**Table 5:** Statistics and official splits of the three fine-grained image classification datasets.

Dataset	Classes	Images	Official Split
CUB-200-2011	200	11,788	Train 5994/Test 5794
Stanford Dogs	120	20,580	Train 12,000/Test 8580
Oxford Flowers-102	102	8189	Train 1020/Val 1020/Test 6149

**Table 6:** Performance and complexity comparison on three fine-grained image classification benchmarks. Top-1 accuracy is reported as mean ± standard deviation.

Model	CUB Top-1 (%)	Dogs Top-1 (%)	Flowers Top-1 (%)	Params (M)	MACs (G)
ResNet-50	81.85 ± 0.10	83.14 ± 0.16	92.80 ± 0.14	23.9	4.09
EfficientNet-B0	79.56 ± 0.41	81.54 ± 0.30	90.53 ± 0.15	4.3	0.39
ConvNeXt-Tiny	77.41 ± 1.03	78.25 ± 0.43	88.42 ± 1.11	28.0	4.46
Swin-Tiny	82.28 ± 0.50	84.15 ± 0.18	93.89 ± 0.14	27.7	4.35
VMamba-Tiny	83.93 ± 0.43	87.60 ± 0.32	94.72 ± 0.78	29.6	4.81
FGBNet	89.10 ± 0.22	89.53 ± 0.13	94.13 ± 0.42	—	—
AMR-VMamba	86.98 ± 0.18	88.51 ± 0.26	96.32 ± 0.23	30.1	4.83

**Table 7:** Component ablation, aggregation-strategy comparison, and MQP query-number sensitivity on CUB-200-2011, Stanford Dogs, and Oxford Flowers-102. D is the default complete model with *K* = 4 and mean aggregation; E uses concat + projection; F1–F4 correspond to *K* = 1, 2, 3, and 5. Results are mean ± sample standard deviation over five runs.

ID	AMR	MQP	*K*	Aggregation	CUB Top-1 (%)	Dogs Top-1 (%)	Flowers Top-1 (%)	Description
A	—	—	—	—	83.93 ± 0.43	87.60 ± 0.32	94.72 ± 0.78	Baseline
B	✓	—	—	—	85.46 ± 0.51	88.06 ± 0.34	95.50 ± 0.52	AMR-only
C	—	✓	4	mean	85.88 ± 0.32	88.18 ± 0.29	95.72 ± 0.44	MQP-only
D	✓	✓	4	mean	86.98 ± 0.18	88.51 ± 0.26	96.32 ± 0.23	Full model
E	✓	✓	4	concat + proj.	85.30 ± 0.41	88.00 ± 0.37	95.41 ± 0.57	Aggregation
F1	✓	✓	1	mean	85.65 ± 0.14	88.10 ± 0.35	95.61 ± 0.49	K sensitivity
F2	✓	✓	2	mean	86.26 ± 0.17	88.29 ± 0.31	95.92 ± 0.36	K sensitivity
F3	✓	✓	3	mean	86.46 ± 0.27	88.36 ± 0.28	96.04 ± 0.31	K sensitivity
F4	✓	✓	5	mean	86.51 ± 0.13	88.39 ± 0.33	96.08 ± 0.34	K sensitivity

**Table 8:** Theoretical complexity and practical inference efficiency of VMamba-Tiny and the enhanced configurations. Latency, FPS, and peak GPU memory are measured for VMamba-Tiny and the complete AMR-VMamba under identical hardware/software settings; dashes indicate values not measured for the intermediate ablation variants.

Model	Params (M)	MACs (G)	ΔParams (M)	ΔMACs (G)	Latency	FPS	Peak GPU Memory
VMamba-Tiny	29.6	4.81	—	—	6.35 ms	157.5	1.72 GB
AMR-only	29.7	4.83	+0.1	+0.02	—	—	—
MQP-only	29.9	4.82	+0.3	+0.01	—	—	—
AMR-VMamba	30.1	4.83	+0.5	+0.02	6.82 ms	146.6	1.81 GB

**Table 9:** CUB-200-2011 performance of the baseline and AMR/MQP-enhanced configurations under two VMamba backbone capacities. Results are mean ± standard deviation over five runs with 95% confidence intervals.

Backbone	Configuration	Mean ± Std	95% CI
VMamba-Tiny	Baseline	83.93 ± 0.43	[83.40, 84.46]
VMamba-Tiny	AMR-VMamba	86.98 ± 0.18	[86.76, 87.20]
VMamba-Small	Baseline	85.74 ± 0.36	[85.29, 86.19]
VMamba-Small	AMR-VMamba	87.62 ± 0.24	[87.32, 87.92]

## Data Availability

The datasets analyzed in this study are publicly available: CUB-200-2011 [16], Stanford Dogs [17], and Oxford Flowers-102 [18]. The official access pages are provided in the corresponding references. The main training settings and optimization hyperparameters are reported in Table 2 and Table 3. The processed datasets, dataset split information, and software source code generated during this study are available from the corresponding author upon reasonable request.

## Abbreviations

The following abbreviations are used in this manuscript:

FGVC	Fine-grained visual classification
AMR	Adaptive multi-level refinement
MQP	Multi-query discriminative pooling

## References

[1] Liu Y., Cai Y., Jia Q., Qiu B., Wang W., Pu N. Novel Class Discovery for Ultra-Fine-Grained Visual Categorization. Proceedings of the IEEE/CVF Conference on Computer Vision and Pattern Recognition (CVPR).

[2] Black S., Souvenir R. Fine-grained Few-Shot Classification with Part Matching. Proceedings of the IEEE/CVF Conference on Computer Vision and Pattern Recognition (CVPR) Workshops.

[3] Jia N., Guo J., Li Y., Tang S., Xu L., Liu L., Xing J. (2024). A fine-grained image classification algorithm based on self-supervised learning and multi-feature fusion of blood cells. Sci. Rep..

[4] Guo Y., Li B., Zhang W., Dong W. (2025). Spatial-frequency feature fusion network for small dataset fine-grained image classification. Sci. Rep..

[5] Yang S., Yang X., Wu J., Feng B. (2024). Significant feature suppression and cross-feature fusion networks for fine-grained visual classification. Sci. Rep..

[6] Gu A., Dao T. Mamba: Linear-Time Sequence Modeling with Selective State Spaces. Proceedings of the First Conference on Language Modeling (COLM).

[7] Liu Y., Tian Y., Zhao Y., Yu H., Xie L., Wang Y., Ye Q., Jiao J., Liu Y. (2024). VMamba: Visual State Space Model. Advances in Neural Information Processing Systems 37, Proceedings of the Thirty-Eighth Annual Conference on Neural Information Processing Systems (NeurIPS 2024), Vancouver, BC, Canada, 10–15 December 2024.

[8] Hatamizadeh A., Kautz J. MambaVision: A Hybrid Mamba-Transformer Vision Backbone. Proceedings of the IEEE/CVF Conference on Computer Vision and Pattern Recognition (CVPR).

[9] Xie F., Wang Z., Zhang W., Ma C. PVMamba: Parallelizing Vision Mamba via Dynamic State Aggregation. Proceedings of the IEEE/CVF International Conference on Computer Vision (ICCV).

[10] Liu L., Zhang M., Yin J., Liu T., Ji W., Piao Y., Lu H. DefMamba: Deformable Visual State Space Model. Proceedings of the IEEE/CVF Conference on Computer Vision and Pattern Recognition (CVPR).

[11] Chen Y., Wang J., Wang P., Zhang R., Li Y. (2025). Vision Mamba Distillation for Low-Resolution Fine-Grained Image Classification. IEEE Signal Process. Lett..

[12] Yuan Y., Huang D., Cai B., Shen Y., Wang J., Xv J., Chen S. (2025). FGBNet: A Bio-Subspecies Classification Network with Multi-Level Feature Interaction. Diversity.

[13] Yu W., Wang X. MambaOut: Do We Really Need Mamba for Vision?. Proceedings of the IEEE/CVF Conference on Computer Vision and Pattern Recognition (CVPR).

[14] Ma X., Ni Z., Chen X. TinyViM: Frequency Decoupling for Tiny Hybrid Vision Mamba. Proceedings of the IEEE/CVF International Conference on Computer Vision (ICCV).

[15] Cubuk E.D., Zoph B., Shlens J., Le Q.V. (2020). RandAugment: Practical Automated Data Augmentation with a Reduced Search Space. Advances in Neural Information Processing Systems 33, Proceedings of the Thirty-Fourth Annual Conference on Neural Information Processing Systems (NeurIPS 2020), Virtual, 6–12 December 2020.

[16] Zhong Z., Zheng L., Kang G., Li S., Yang Y. (2020). Random Erasing Data Augmentation. Proc. AAAI Conf. Artif. Intell..

[17] Wah C., Branson S., Welinder P., Perona P., Belongie S. (2011). The Caltech-UCSD Birds-200-2011 Dataset.

[18] Khosla A., Jayadevaprakash N., Yao B., Fei-Fei L. Novel Dataset for Fine-Grained Image Categorization: Stanford Dogs. Proceedings of the CVPR Workshop on Fine-Grained Visual Categorization (FGVC).

[19] Nilsback M.-E., Zisserman A. Automated Flower Classification over a Large Number of Classes. Proceedings of the 2008 Sixth Indian Conference on Computer Vision, Graphics & Image Processing (ICVGIP).

[20] He K., Zhang X., Ren S., Sun J. Deep Residual Learning for Image Recognition. Proceedings of the IEEE Conference on Computer Vision and Pattern Recognition (CVPR).

[21] Tan M., Le Q.V. (2019). EfficientNet: Rethinking Model Scaling for Convolutional Neural Networks. Proceedings of the 36th International Conference on Machine Learning (ICML), Long Beach, CA, USA, 9–15 June 2019.

[22] Liu Z., Mao H., Wu C.-Y., Feichtenhofer C., Darrell T., Xie S. A ConvNet for the 2020s. Proceedings of the IEEE/CVF Conference on Computer Vision and Pattern Recognition (CVPR).

[23] Liu Z., Lin Y., Cao Y., Hu H., Wei Y., Zhang Z., Lin S., Guo B. Swin Transformer: Hierarchical Vision Transformer Using Shifted Windows. Proceedings of the IEEE/CVF International Conference on Computer Vision (ICCV).

